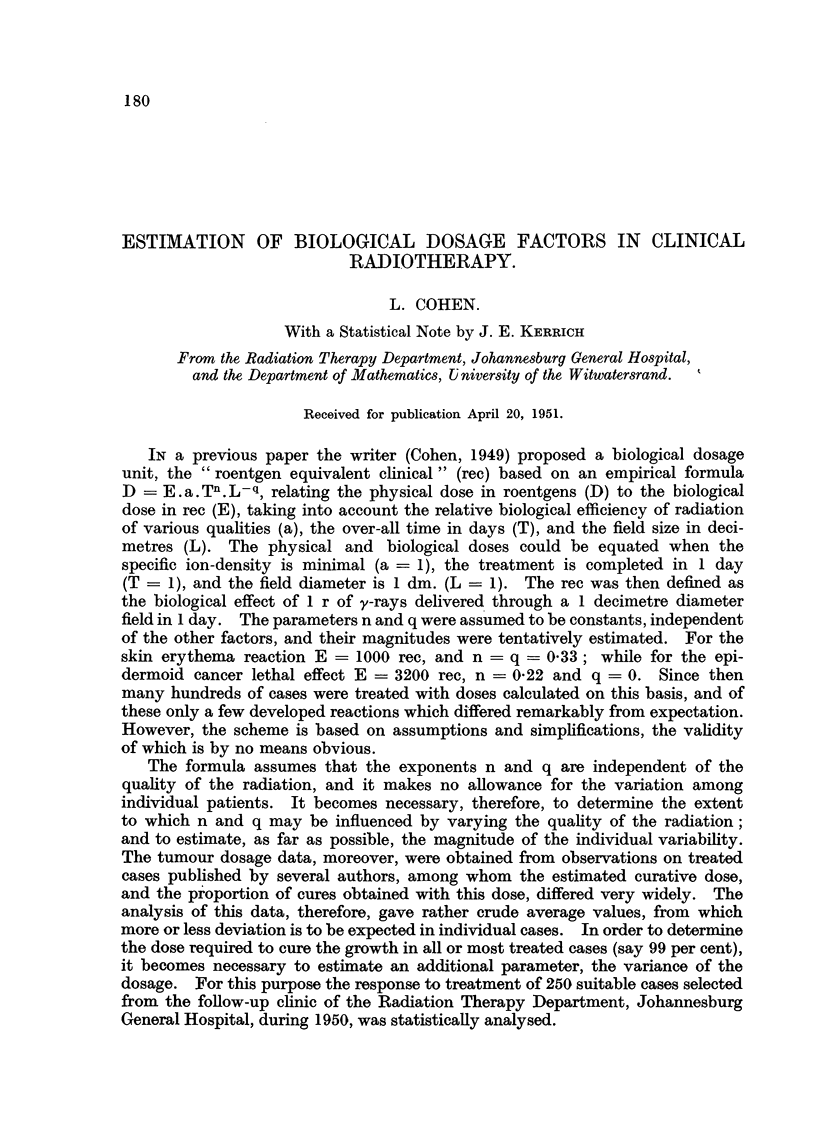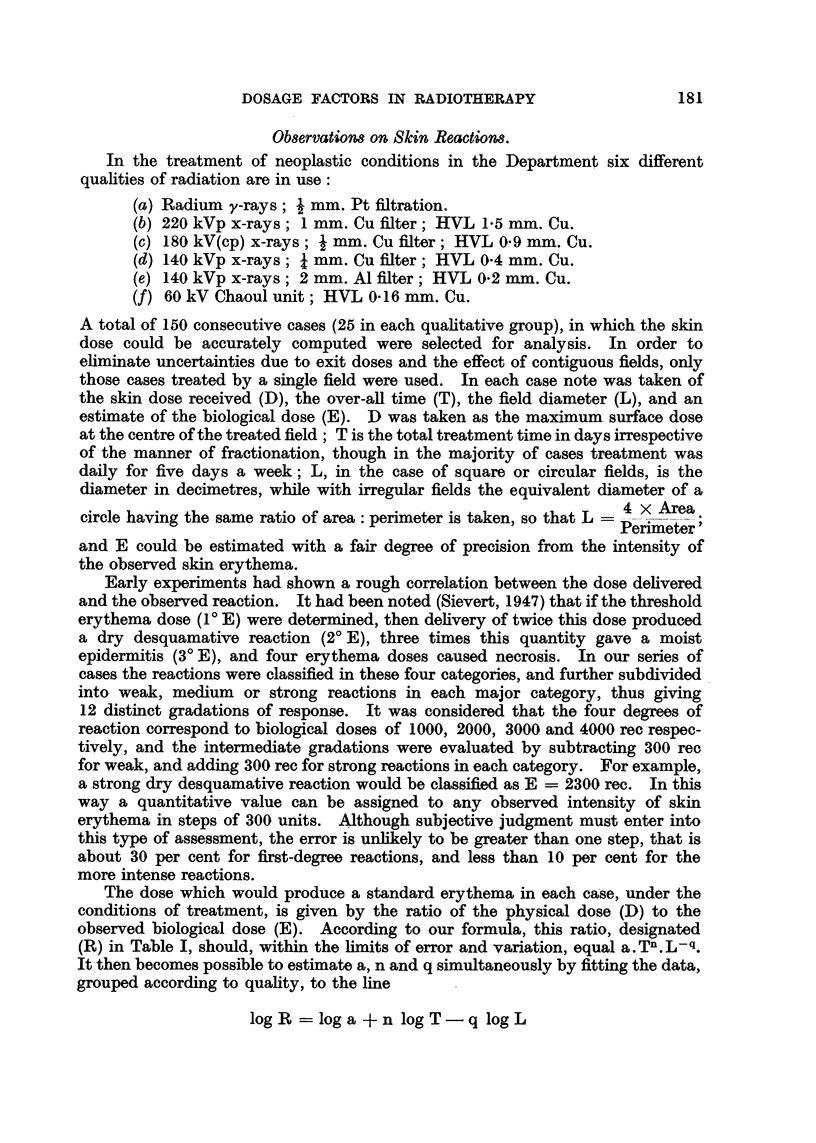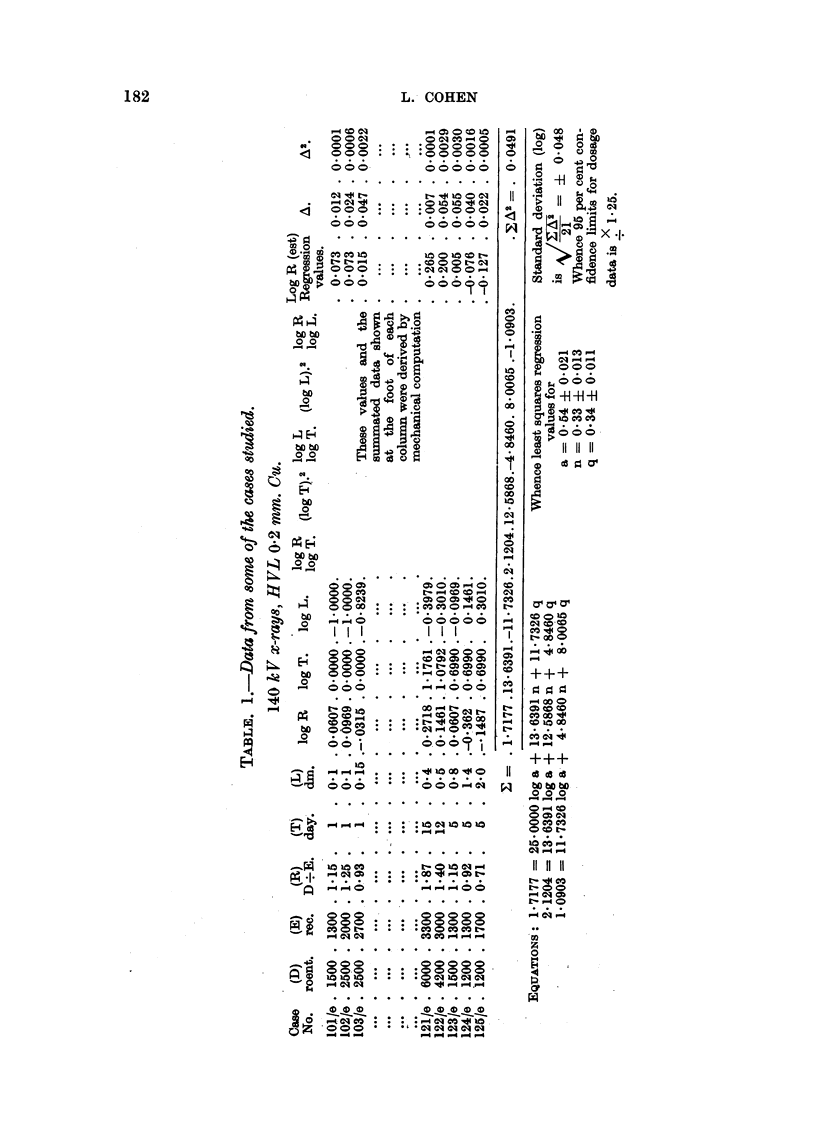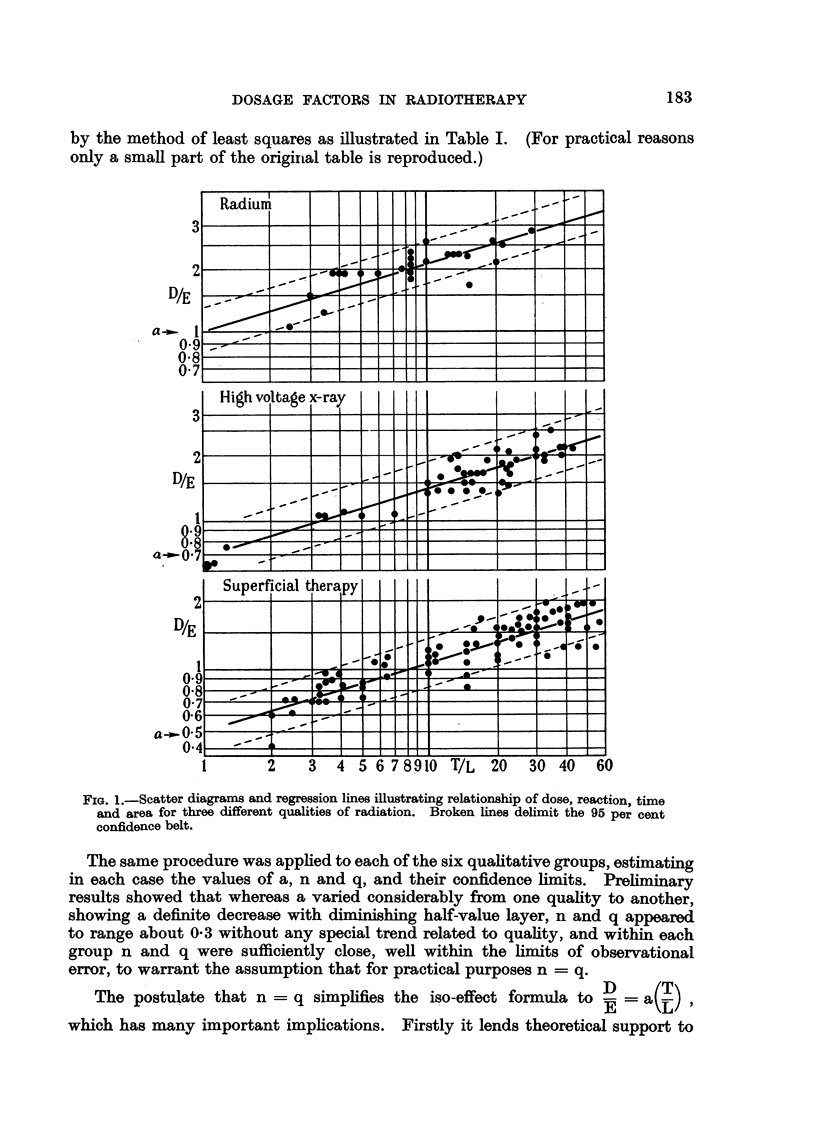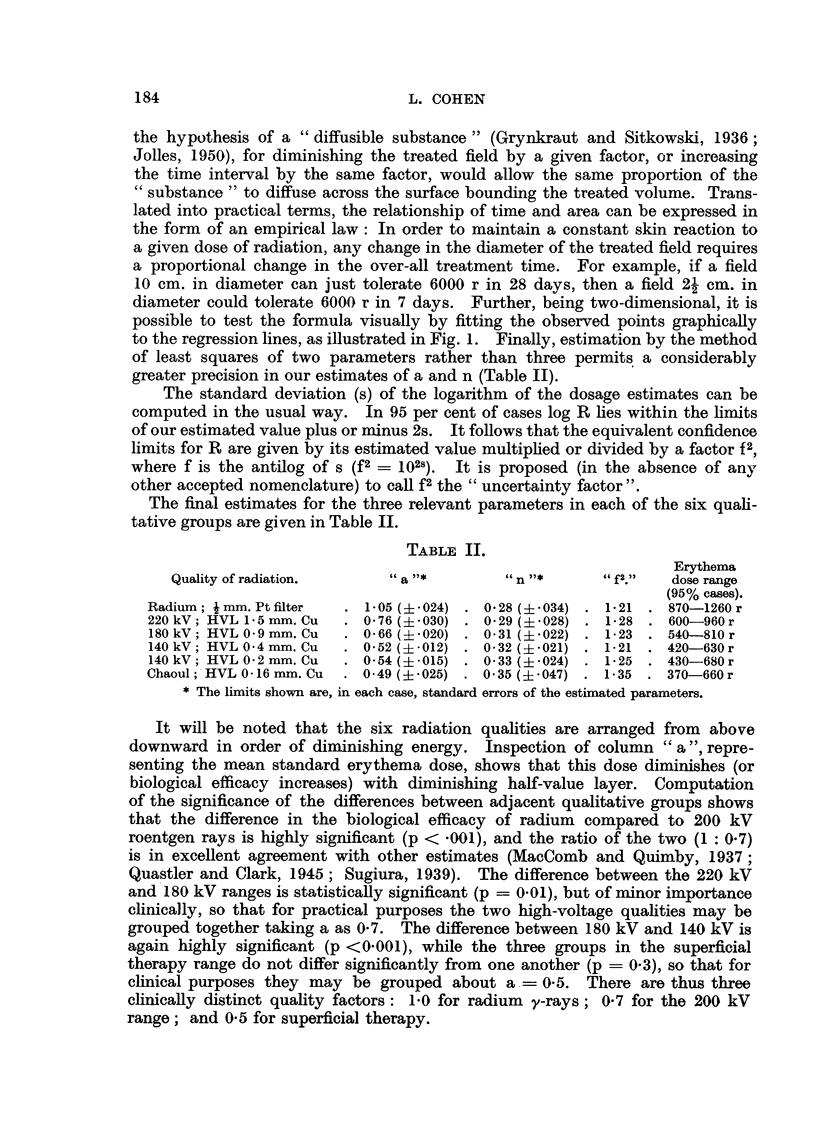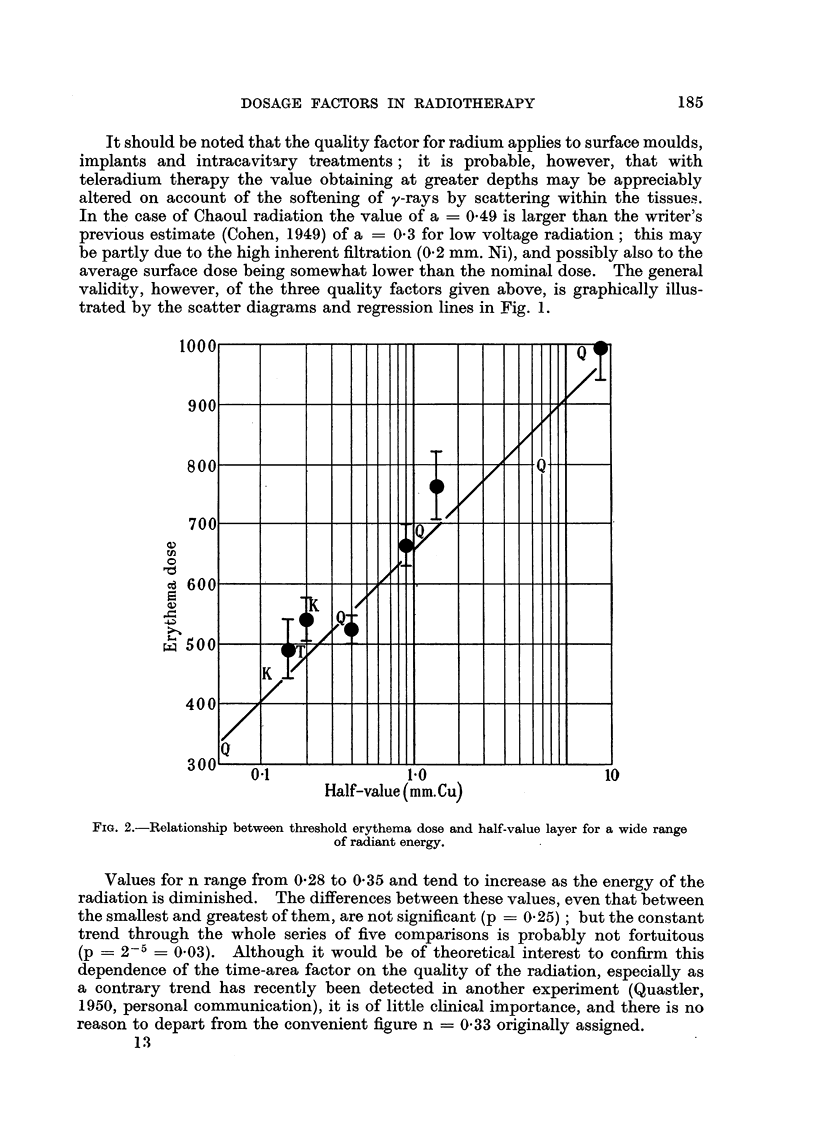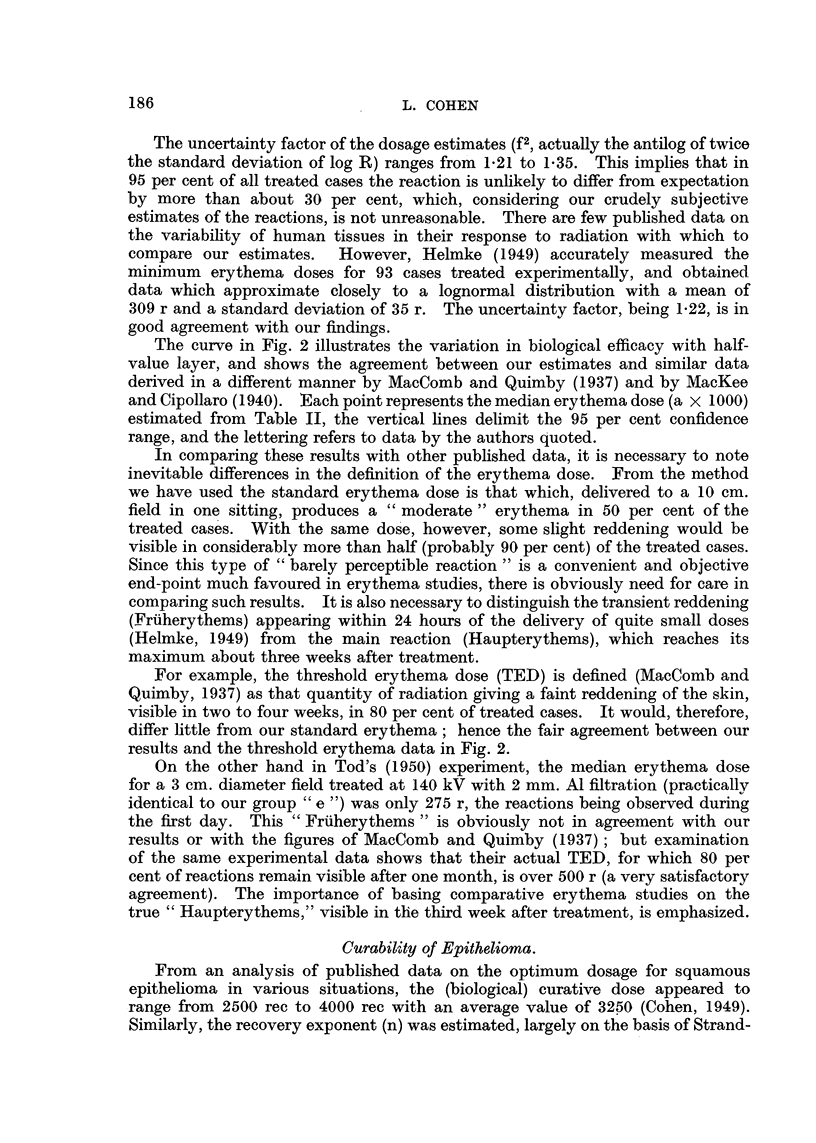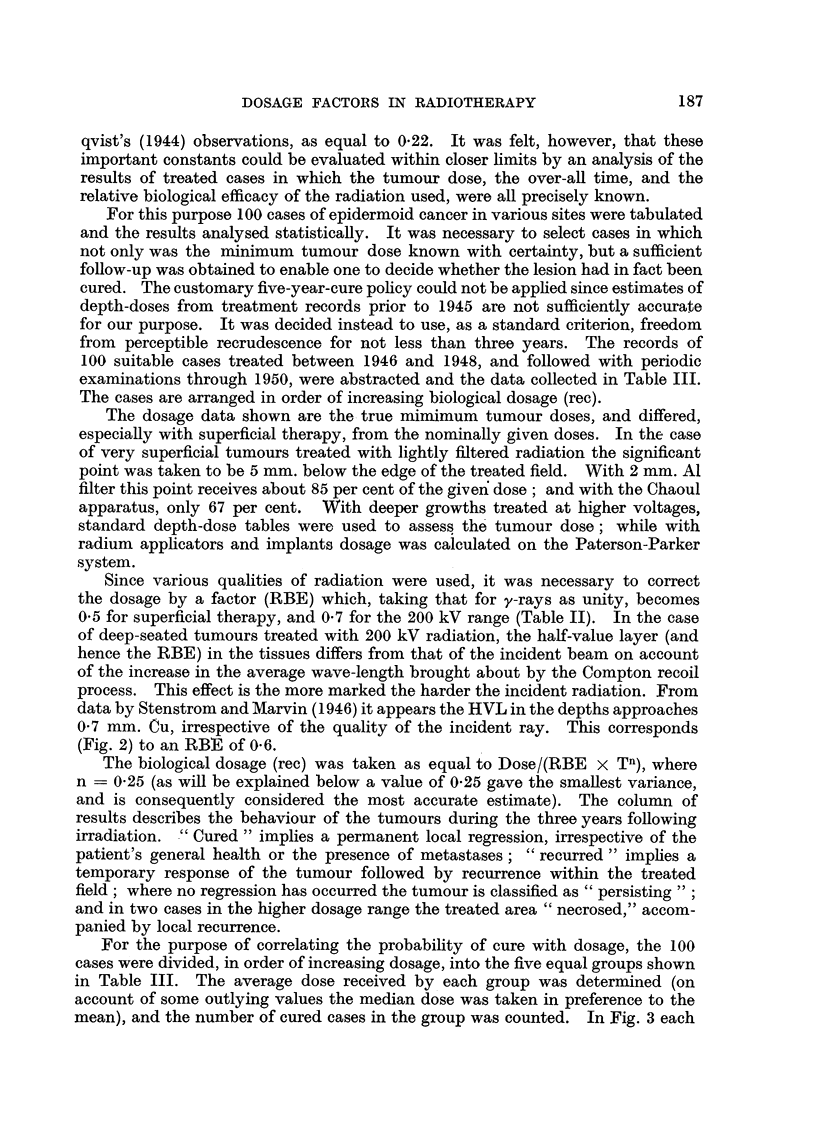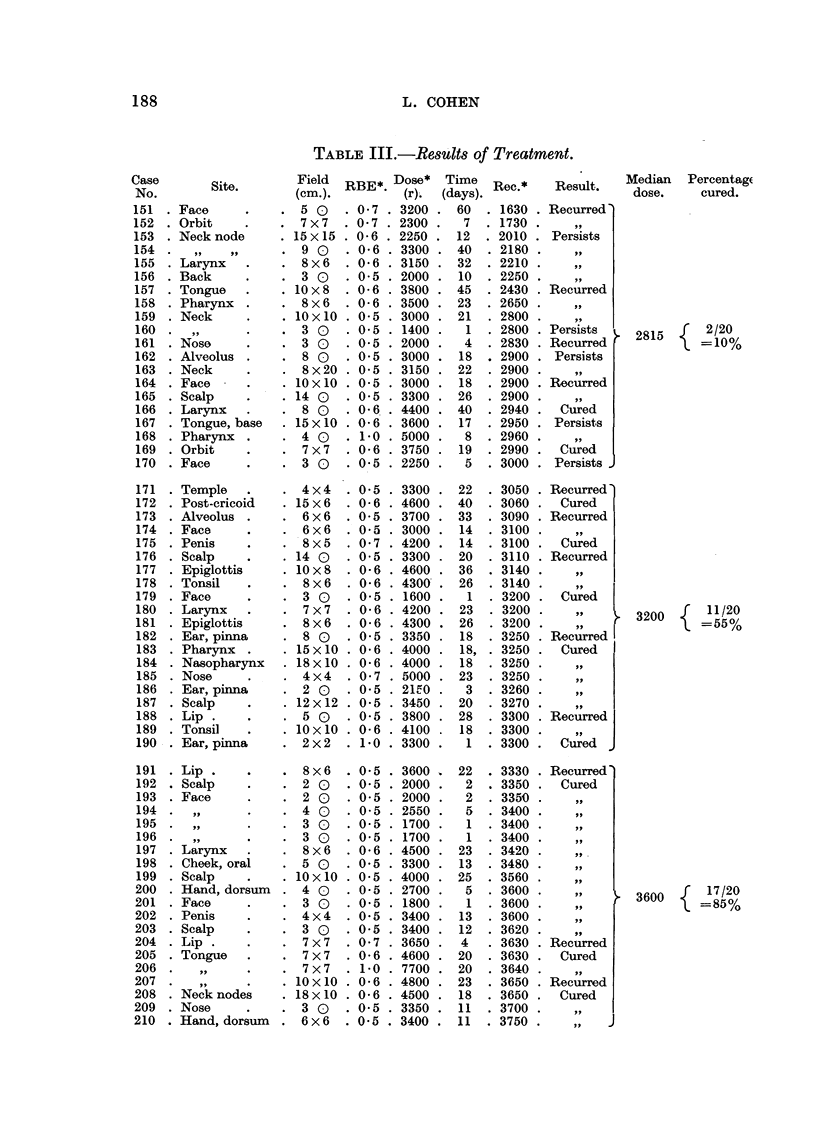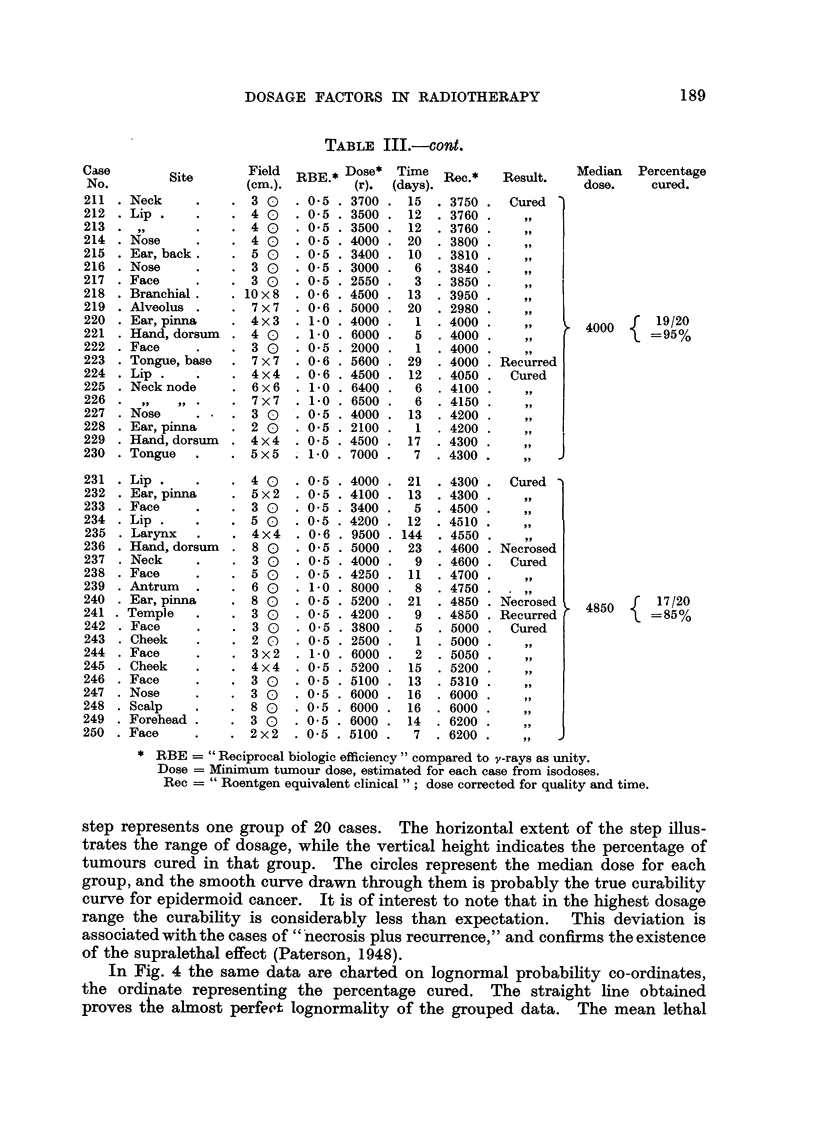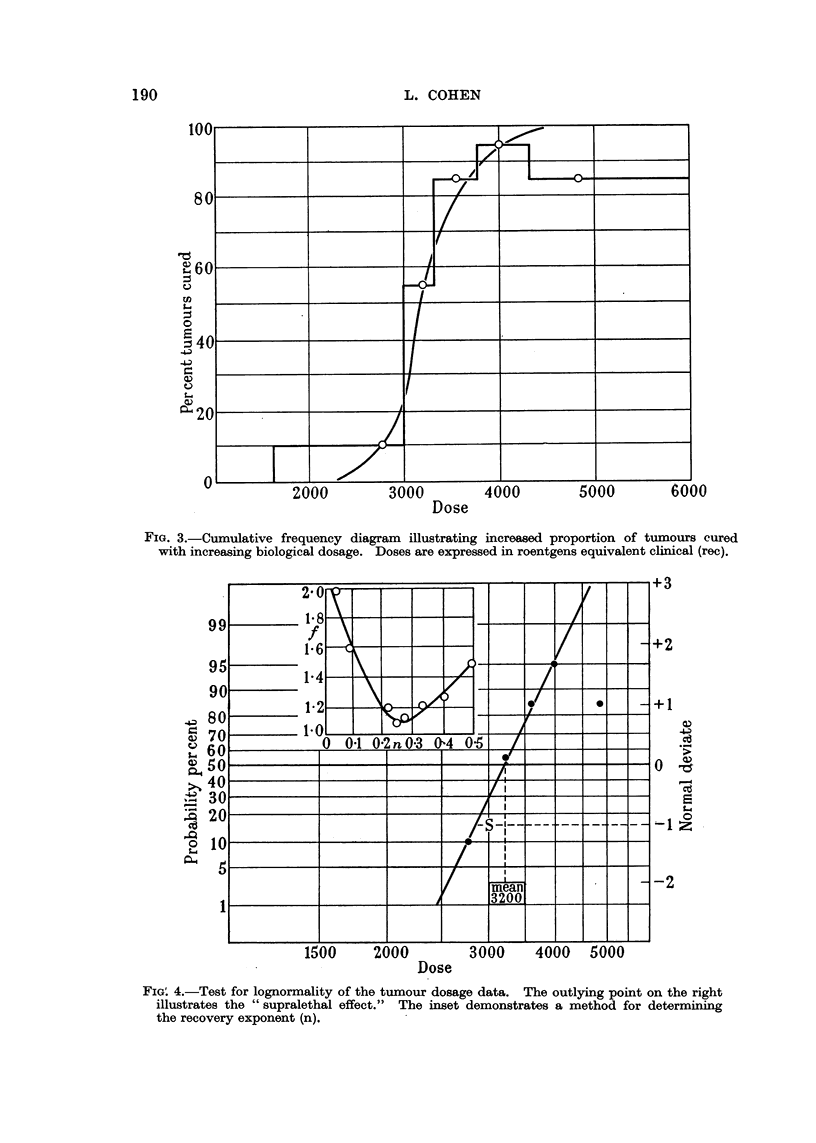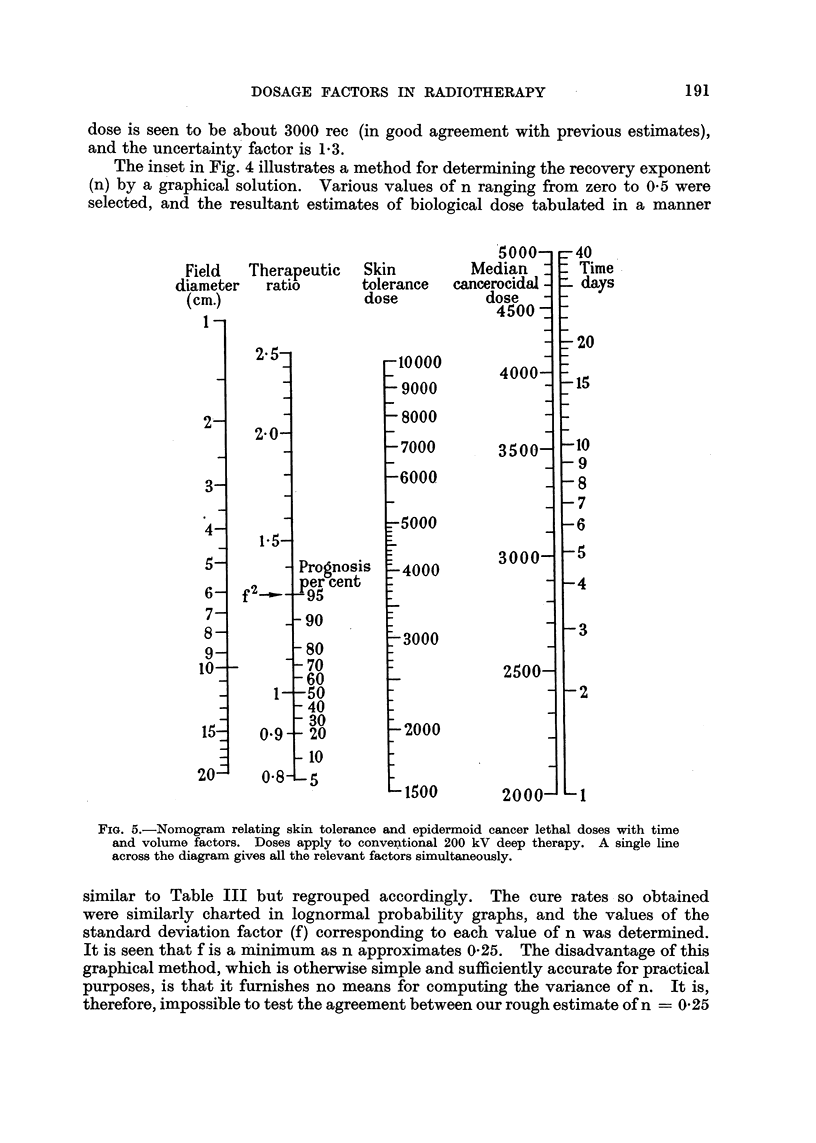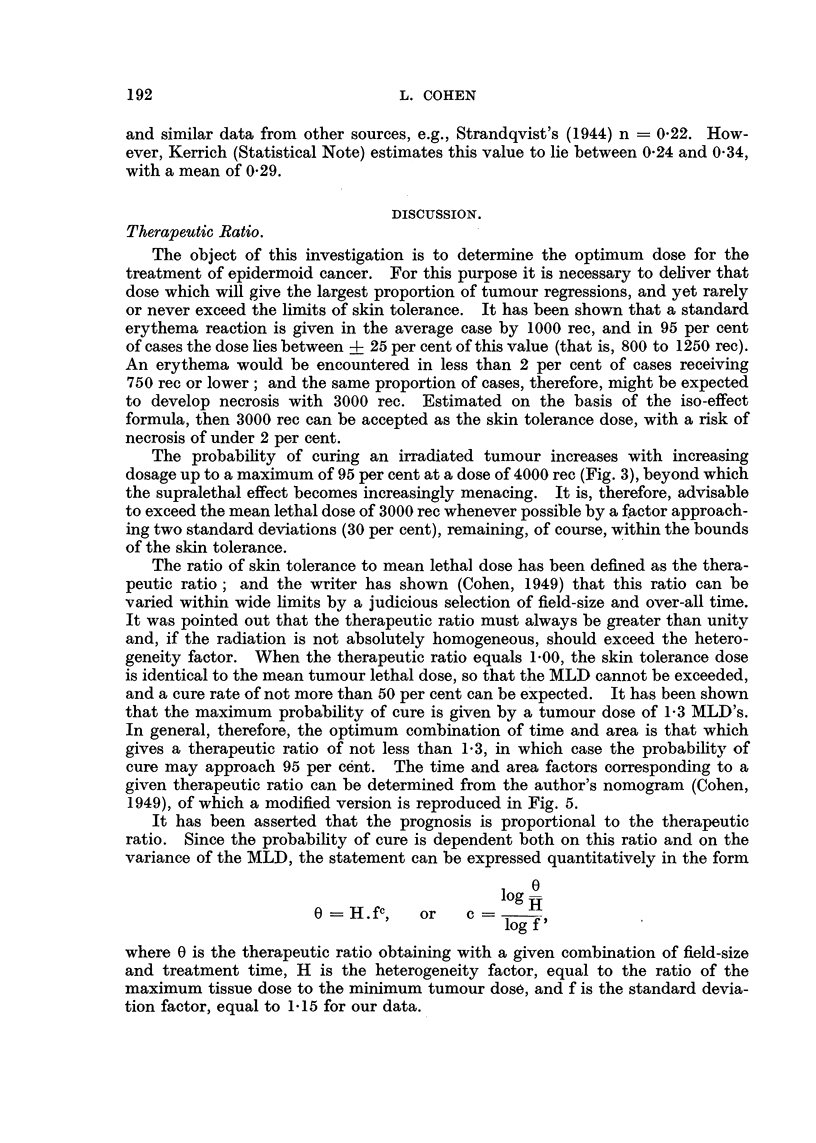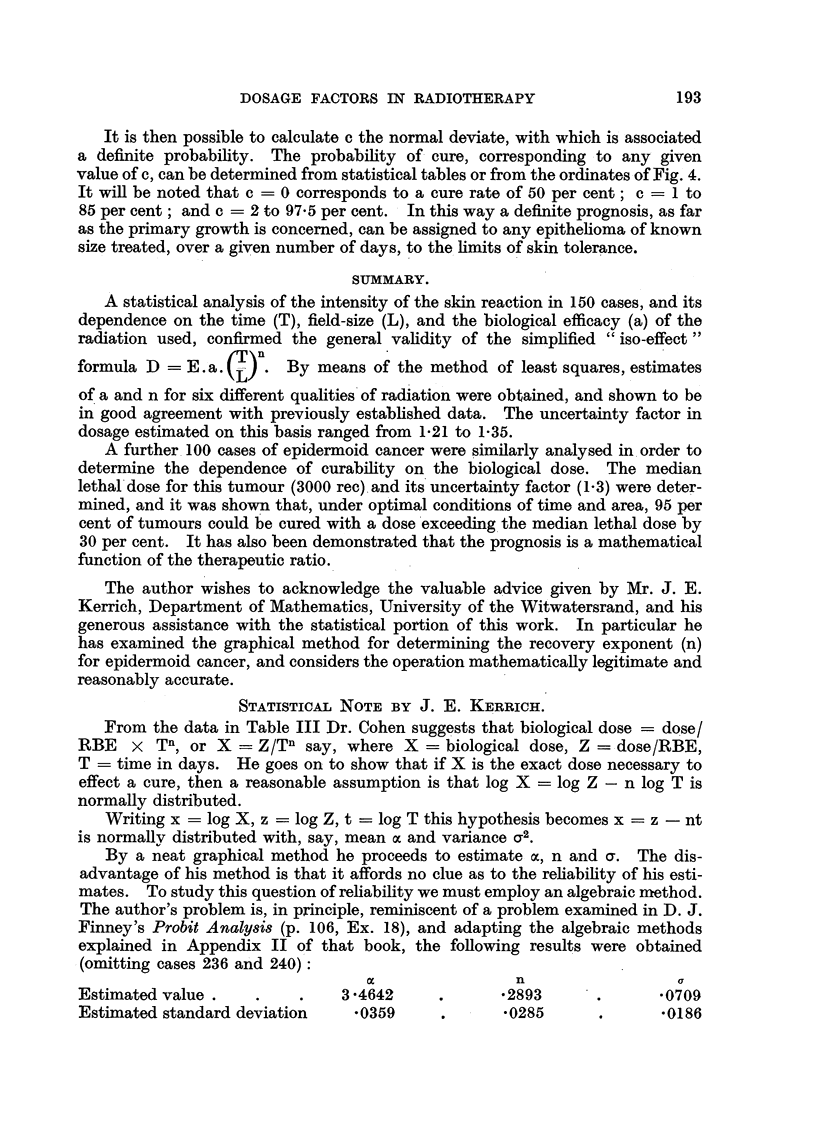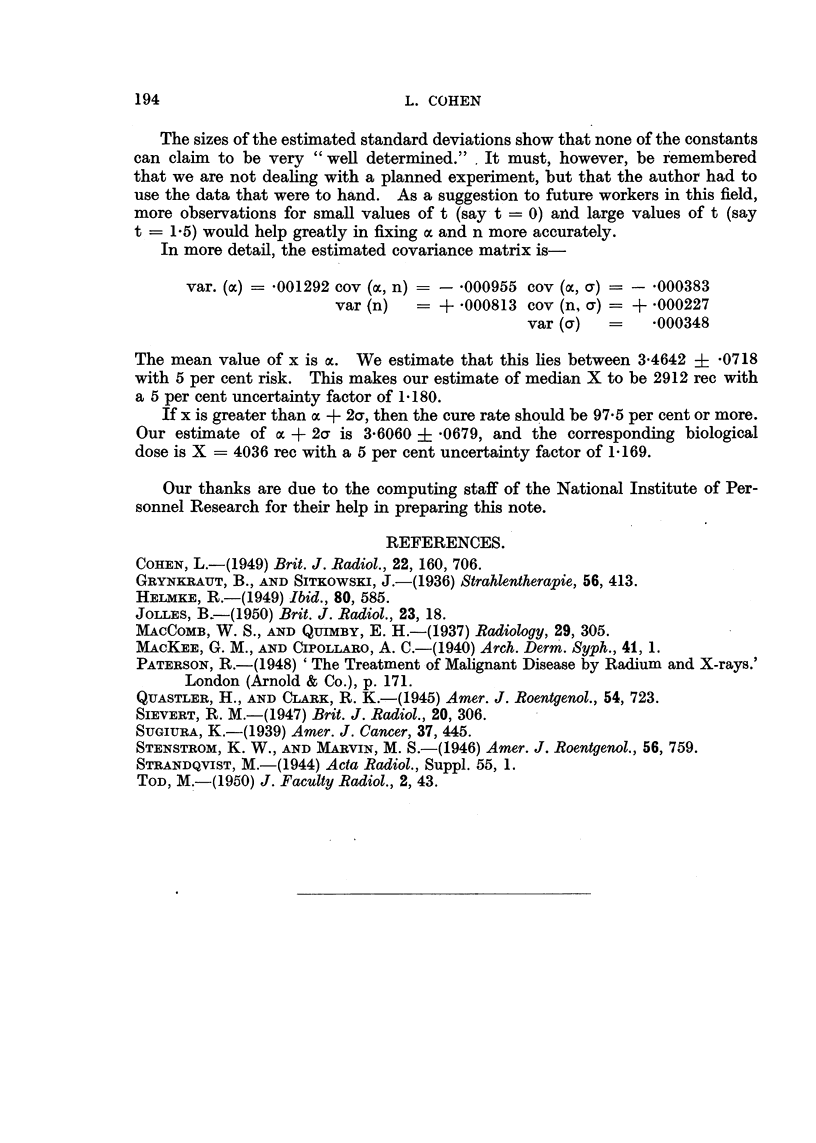# Estimation of Biological Dosage Factors in Clinical Radiotherapy

**DOI:** 10.1038/bjc.1951.20

**Published:** 1951-06

**Authors:** L. Cohen


### ESTIMATION OF BIOLOGICAL DOSAGE FACTORS IN CLINICAL RADIOTHERAPY.

L. COHEN.

With a Statistical Note by J. E. KERRICH

*From the Radiation Therapy Department, Johannesburg General Hospital, and the Department of Mathematics, University of the Witwatersrand.*

Received for publication April 20, 1951.

IN a previous paper the writer (Cohen, 1949) proposed a biological dosage unit, the "roentgen equivalent clinical" (rec) based on an empirical formula $D = E.a.T^n.L^{-q}$, relating the physical dose in roentgens (D) to the biological dose in rec (E), taking into account the relative biological efficiency of radiation of various qualities (a), the over-all time in days (T), and the field size in decimetres (L). The physical and biological doses could be equated when the specific ion-density is minimal (a = 1), the treatment is completed in 1 day (T = 1), and the field diameter is 1 dm. (L = 1). The rec was then defined as the biological effect of 1 r of $\gamma$-rays delivered through a 1 decimetre diameter field in 1 day. The parameters n and q were assumed to be constants, independent of the other factors, and their magnitudes were tentatively estimated. For the skin erythema reaction E = 1000 rec, and n = q = 0·33 ; while for the epidermoid cancer lethal effect E = 3200 rec, n = 0·22 and q = 0. Since then many hundreds of cases were treated with doses calculated on this basis, and of these only a few developed reactions which differed remarkably from expectation. However, the scheme is based on assumptions and simplifications, the validity of which is by no means obvious.

The formula assumes that the exponents n and q are independent of the quality of the radiation, and it makes no allowance for the variation among individual patients. It becomes necessary, therefore, to determine the extent to which n and q may be influenced by varying the quality of the radiation ; and to estimate, as far as possible, the magnitude of the individual variability. The tumour dosage data, moreover, were obtained from observations on treated cases published by several authors, among whom the estimated curative dose, and the proportion of cures obtained with this dose, differed very widely. The analysis of this data, therefore, gave rather crude average values, from which more or less deviation is to be expected in individual cases. In order to determine the dose required to cure the growth in all or most treated cases (say 99 per cent), it becomes necessary to estimate an additional parameter, the variance of the dosage. For this purpose the response to treatment of 250 suitable cases selected from the follow-up clinic of the Radiation Therapy Department, Johannesburg General Hospital, during 1950, was statistically analysed.

*Observations on Skin Reactions.*

In the treatment of neoplastic conditions in the Department six different qualities of radiation are in use :

(*a*) Radium $\gamma$-rays ; $\frac{1}{2}$ mm. Pt filtration.

(*b*) 220 kVp x-rays ; 1 mm. Cu filter ; HVL 1·5 mm. Cu.

(*c*) 180 kV(cp) x-rays ; $\frac{1}{2}$ mm. Cu filter ; HVL 0·9 mm. Cu.

(*d*) 140 kVp x-rays ; $\frac{1}{4}$ mm. Cu filter ; HVL 0·4 mm. Cu.

(*e*) 140 kVp x-rays ; 2 mm. Al filter ; HVL 0·2 mm. Cu.

(*f*) 60 kV Chaoul unit ; HVL 0·16 mm. Cu.

A total of 150 consecutive cases (25 in each qualitative group), in which the skin dose could be accurately computed were selected for analysis. In order to eliminate uncertainties due to exit doses and the effect of contiguous fields, only those cases treated by a single field were used. In each case note was taken of the skin dose received (D), the over-all time (T), the field diameter (L), and an estimate of the biological dose (E). D was taken as the maximum surface dose at the centre of the treated field ; T is the total treatment time in days irrespective of the manner of fractionation, though in the majority of cases treatment was daily for five days a week ; L, in the case of square or circular fields, is the diameter in decimetres, while with irregular fields the equivalent diameter of a circle having the same ratio of area : perimeter is taken, so that $L = \frac{4 \times \text{Area}}{\text{Perimeter}}$; and E could be estimated with a fair degree of precision from the intensity of the observed skin erythema.

Early experiments had shown a rough correlation between the dose delivered and the observed reaction. It had been noted (Sievert, 1947) that if the threshold erythema dose (1° E) were determined, then delivery of twice this dose produced a dry desquamative reaction (2° E), three times this quantity gave a moist epidermitis (3° E), and four erythema doses caused necrosis. In our series of cases the reactions were classified in these four categories, and further subdivided into weak, medium or strong reactions in each major category, thus giving 12 distinct gradations of response. It was considered that the four degrees of reaction correspond to biological doses of 1000, 2000, 3000 and 4000 rec respectively, and the intermediate gradations were evaluated by subtracting 300 rec for weak, and adding 300 rec for strong reactions in each category. For example, a strong dry desquamative reaction would be classified as E = 2300 rec. In this way a quantitative value can be assigned to any observed intensity of skin erythema in steps of 300 units. Although subjective judgment must enter into this type of assessment, the error is unlikely to be greater than one step, that is about 30 per cent for first-degree reactions, and less than 10 per cent for the more intense reactions.

The dose which would produce a standard erythema in each case, under the conditions of treatment, is given by the ratio of the physical dose (D) to the observed biological dose (E). According to our formula, this ratio, designated (R) in Table I, should, within the limits of error and variation, equal $a.T^{n}.L^{-q}$. It then becomes possible to estimate a, n and q simultaneously by fitting the data, grouped according to quality, to the line

$$\log R = \log a + n \log T - q \log L$$

TABLE. 1.—*Data from some of the cases studied.*

140 *kV x-rays, HVL* 0·2 *mm. Cu.*

| Case No. | (D) roent. | (E) rec. | (R) D÷E. | (T) day. | (L) dm. | log R | log T. | log L. | log R log T. | (log T).² | log L log T. | (log L).² | log R log L. | Log R (est) Regression values. | Δ. | Δ². |
|---|---|---|---|---|---|---|---|---|---|---|---|---|---|---|---|---|
| 101/e | 1500 | 1300 | 1·15 | 1 | 0·1 | 0·0607 | 0·0000 | −1·0000 | | | | | | 0·073 | 0·012 | 0·0001 |
| 102/e | 2500 | 2000 | 1·25 | 1 | 0·1 | 0·0969 | 0·0000 | −1·0000 | | | | | | 0·073 | 0·024 | 0·0006 |
| 103/e | 2500 | 2700 | 0·93 | 1 | 0·15 | −·0315 | 0·0000 | −0·8239 | | | These values and the | | | 0·015 | 0·047 | 0·0022 |
| ... | ... | ... | ... | ... | ... | ... | ... | ... | | | summated data shown | | | ... | ... | ... |
| ... | ... | ... | ... | ... | ... | ... | ... | ... | | | at the foot of each | | | ... | ... | ... |
| ... | ... | ... | ... | ... | ... | ... | ... | ... | | | column were derived by | | | ... | ... | ... |
| ... | ... | ... | ... | ... | ... | ... | ... | ... | | | mechanical computation | | | ... | ... | ... |
| 121/e | 6000 | 3300 | 1·87 | 15 | 0·4 | 0·2718 | 1·1761 | −0·3979 | | | | | | 0·265 | 0·007 | 0·0001 |
| 122/e | 4200 | 3000 | 1·40 | 12 | 0·5 | 0·1461 | 1·0792 | −0·3010 | | | | | | 0·200 | 0·054 | 0·0029 |
| 123/e | 1500 | 1300 | 1·15 | 5 | 0·8 | 0·0607 | 0·6990 | −0·0969 | | | | | | 0·005 | 0·055 | 0·0030 |
| 124/e | 1200 | 1300 | 0·92 | 5 | 1·4 | −0·362 | 0·6990 | 0·1461 | | | | | | −0·076 | 0·040 | 0·0016 |
| 125/e | 1200 | 1700 | 0·71 | 5 | 2·0 | −·1487 | 0·6990 | 0·3010 | | | | | | −0·127 | 0·022 | 0·0005 |
| | | | | | Σ = | 1·7177 | 13·6391 | −11·7326 | 2·1204 | 12·5868 | −4·8460 | 8·0065 | −1·0903 | | ΣΔ² = | 0·0491 |

EQUATIONS: $1{\cdot}7177 = 25{\cdot}0000 \log a + 13{\cdot}6391\, n + 11{\cdot}7326\, q$

$2{\cdot}1204 = 13{\cdot}6391 \log a + 12{\cdot}5868\, n + 4{\cdot}8460\, q$

$1{\cdot}0903 = 11{\cdot}7326 \log a + 4{\cdot}8460\, n + 8{\cdot}0065\, q$

Whence least squares regression values for

$a = 0{\cdot}54 \pm 0{\cdot}021$

$n = 0{\cdot}33 \pm 0{\cdot}013$

$q = 0{\cdot}34 \pm 0{\cdot}011$

Standard deviation (log) is $\sqrt{\frac{\Sigma\Delta^2}{21}} = \pm\ 0{\cdot}048$

Whence 95 per cent confidence limits for dosage data is $\overset{\times}{\div}\ 1{\cdot}25$.

by the method of least squares as illustrated in Table I. (For practical reasons only a small part of the original table is reproduced.)

FIG. 1.—Scatter diagrams and regression lines illustrating relationship of dose, reaction, time and area for three different qualities of radiation. Broken lines delimit the 95 per cent confidence belt.

The same procedure was applied to each of the six qualitative groups, estimating in each case the values of a, n and q, and their confidence limits. Preliminary results showed that whereas a varied considerably from one quality to another, showing a definite decrease with diminishing half-value layer, n and q appeared to range about 0·3 without any special trend related to quality, and within each group n and q were sufficiently close, well within the limits of observational error, to warrant the assumption that for practical purposes n = q.

The postulate that n = q simplifies the iso-effect formula to $\frac{D}{E} = a\left(\frac{T}{L}\right)$, which has many important implications. Firstly it lends theoretical support to the hypothesis of a " diffusible substance " (Grynkraut and Sitkowski, 1936; Jolles, 1950), for diminishing the treated field by a given factor, or increasing the time interval by the same factor, would allow the same proportion of the " substance " to diffuse across the surface bounding the treated volume. Translated into practical terms, the relationship of time and area can be expressed in the form of an empirical law: In order to maintain a constant skin reaction to a given dose of radiation, any change in the diameter of the treated field requires a proportional change in the over-all treatment time. For example, if a field 10 cm. in diameter can just tolerate 6000 r in 28 days, then a field $2\frac{1}{2}$ cm. in diameter could tolerate 6000 r in 7 days. Further, being two-dimensional, it is possible to test the formula visually by fitting the observed points graphically to the regression lines, as illustrated in Fig. 1. Finally, estimation by the method of least squares of two parameters rather than three permits a considerably greater precision in our estimates of a and n (Table II).

The standard deviation (s) of the logarithm of the dosage estimates can be computed in the usual way. In 95 per cent of cases log R lies within the limits of our estimated value plus or minus 2s. It follows that the equivalent confidence limits for R are given by its estimated value multiplied or divided by a factor $f^2$, where f is the antilog of s ($f^2 = 10^{2s}$). It is proposed (in the absence of any other accepted nomenclature) to call $f^2$ the " uncertainty factor ".

The final estimates for the three relevant parameters in each of the six qualitative groups are given in Table II.

TABLE II.

| Quality of radiation. | " a "* | " n "* | " $f^2$." | Erythema dose range (95% cases). |
|---|---|---|---|---|
| Radium; $\frac{1}{2}$ mm. Pt filter | 1·05 (±·024) | 0·28 (±·034) | 1·21 | 870—1260 r |
| 220 kV; HVL 1·5 mm. Cu | 0·76 (±·030) | 0·29 (±·028) | 1·28 | 600—960 r |
| 180 kV; HVL 0·9 mm. Cu | 0·66 (±·020) | 0·31 (±·022) | 1·23 | 540—810 r |
| 140 kV; HVL 0·4 mm. Cu | 0·52 (±·012) | 0·32 (±·021) | 1·21 | 420—630 r |
| 140 kV; HVL 0·2 mm. Cu | 0·54 (±·015) | 0·33 (±·024) | 1·25 | 430—680 r |
| Chaoul; HVL 0·16 mm. Cu | 0·49 (±·025) | 0·35 (±·047) | 1·35 | 370—660 r |

* The limits shown are, in each case, standard errors of the estimated parameters.

It will be noted that the six radiation qualities are arranged from above downward in order of diminishing energy. Inspection of column " a ", representing the mean standard erythema dose, shows that this dose diminishes (or biological efficacy increases) with diminishing half-value layer. Computation of the significance of the differences between adjacent qualitative groups shows that the difference in the biological efficacy of radium compared to 200 kV roentgen rays is highly significant ($p < \cdot 001$), and the ratio of the two (1 : 0·7) is in excellent agreement with other estimates (MacComb and Quimby, 1937; Quastler and Clark, 1945; Sugiura, 1939). The difference between the 220 kV and 180 kV ranges is statistically significant ($p = 0{\cdot}01$), but of minor importance clinically, so that for practical purposes the two high-voltage qualities may be grouped together taking a as 0·7. The difference between 180 kV and 140 kV is again highly significant ($p < 0{\cdot}001$), while the three groups in the superficial therapy range do not differ significantly from one another ($p = 0{\cdot}3$), so that for clinical purposes they may be grouped about $a = 0{\cdot}5$. There are thus three clinically distinct quality factors: 1·0 for radium $\gamma$-rays; 0·7 for the 200 kV range; and 0·5 for superficial therapy.

It should be noted that the quality factor for radium applies to surface moulds, implants and intracavitary treatments; it is probable, however, that with teleradium therapy the value obtaining at greater depths may be appreciably altered on account of the softening of $\gamma$-rays by scattering within the tissues. In the case of Chaoul radiation the value of a = 0·49 is larger than the writer's previous estimate (Cohen, 1949) of a = 0·3 for low voltage radiation; this may be partly due to the high inherent filtration (0·2 mm. Ni), and possibly also to the average surface dose being somewhat lower than the nominal dose. The general validity, however, of the three quality factors given above, is graphically illustrated by the scatter diagrams and regression lines in Fig. 1.

Fig. 2.—Relationship between threshold erythema dose and half-value layer for a wide range of radiant energy.

Values for n range from 0·28 to 0·35 and tend to increase as the energy of the radiation is diminished. The differences between these values, even that between the smallest and greatest of them, are not significant (p = 0·25); but the constant trend through the whole series of five comparisons is probably not fortuitous ($p = 2^{-5} = 0{\cdot}03$). Although it would be of theoretical interest to confirm this dependence of the time-area factor on the quality of the radiation, especially as a contrary trend has recently been detected in another experiment (Quastler, 1950, personal communication), it is of little clinical importance, and there is no reason to depart from the convenient figure n = 0·33 originally assigned.

The uncertainty factor of the dosage estimates ($f^2$, actually the antilog of twice the standard deviation of log R) ranges from 1·21 to 1·35. This implies that in 95 per cent of all treated cases the reaction is unlikely to differ from expectation by more than about 30 per cent, which, considering our crudely subjective estimates of the reactions, is not unreasonable. There are few published data on the variability of human tissues in their response to radiation with which to compare our estimates. However, Helmke (1949) accurately measured the minimum erythema doses for 93 cases treated experimentally, and obtained data which approximate closely to a lognormal distribution with a mean of 309 r and a standard deviation of 35 r. The uncertainty factor, being 1·22, is in good agreement with our findings.

The curve in Fig. 2 illustrates the variation in biological efficacy with half-value layer, and shows the agreement between our estimates and similar data derived in a different manner by MacComb and Quimby (1937) and by MacKee and Cipollaro (1940). Each point represents the median erythema dose (a $\times$ 1000) estimated from Table II, the vertical lines delimit the 95 per cent confidence range, and the lettering refers to data by the authors quoted.

In comparing these results with other published data, it is necessary to note inevitable differences in the definition of the erythema dose. From the method we have used the standard erythema dose is that which, delivered to a 10 cm. field in one sitting, produces a " moderate " erythema in 50 per cent of the treated cases. With the same dose, however, some slight reddening would be visible in considerably more than half (probably 90 per cent) of the treated cases. Since this type of " barely perceptible reaction " is a convenient and objective end-point much favoured in erythema studies, there is obviously need for care in comparing such results. It is also necessary to distinguish the transient reddening (Früherythems) appearing within 24 hours of the delivery of quite small doses (Helmke, 1949) from the main reaction (Haupterythems), which reaches its maximum about three weeks after treatment.

For example, the threshold erythema dose (TED) is defined (MacComb and Quimby, 1937) as that quantity of radiation giving a faint reddening of the skin, visible in two to four weeks, in 80 per cent of treated cases. It would, therefore, differ little from our standard erythema ; hence the fair agreement between our results and the threshold erythema data in Fig. 2.

On the other hand in Tod's (1950) experiment, the median erythema dose for a 3 cm. diameter field treated at 140 kV with 2 mm. Al filtration (practically identical to our group " e ") was only 275 r, the reactions being observed during the first day. This " Früherythems " is obviously not in agreement with our results or with the figures of MacComb and Quimby (1937) ; but examination of the same experimental data shows that their actual TED, for which 80 per cent of reactions remain visible after one month, is over 500 r (a very satisfactory agreement). The importance of basing comparative erythema studies on the true " Haupterythems," visible in the third week after treatment, is emphasized.

*Curability of Epithelioma.*

From an analysis of published data on the optimum dosage for squamous epithelioma in various situations, the (biological) curative dose appeared to range from 2500 rec to 4000 rec with an average value of 3250 (Cohen, 1949). Similarly, the recovery exponent (n) was estimated, largely on the basis of Strandqvist's (1944) observations, as equal to 0·22. It was felt, however, that these important constants could be evaluated within closer limits by an analysis of the results of treated cases in which the tumour dose, the over-all time, and the relative biological efficacy of the radiation used, were all precisely known.

For this purpose 100 cases of epidermoid cancer in various sites were tabulated and the results analysed statistically. It was necessary to select cases in which not only was the minimum tumour dose known with certainty, but a sufficient follow-up was obtained to enable one to decide whether the lesion had in fact been cured. The customary five-year-cure policy could not be applied since estimates of depth-doses from treatment records prior to 1945 are not sufficiently accurate for our purpose. It was decided instead to use, as a standard criterion, freedom from perceptible recrudescence for not less than three years. The records of 100 suitable cases treated between 1946 and 1948, and followed with periodic examinations through 1950, were abstracted and the data collected in Table III. The cases are arranged in order of increasing biological dosage (rec).

The dosage data shown are the true mimimum tumour doses, and differed, especially with superficial therapy, from the nominally given doses. In the case of very superficial tumours treated with lightly filtered radiation the significant point was taken to be 5 mm. below the edge of the treated field. With 2 mm. Al filter this point receives about 85 per cent of the given dose ; and with the Chaoul apparatus, only 67 per cent. With deeper growths treated at higher voltages, standard depth-dose tables were used to assess the tumour dose ; while with radium applicators and implants dosage was calculated on the Paterson-Parker system.

Since various qualities of radiation were used, it was necessary to correct the dosage by a factor (RBE) which, taking that for $\gamma$-rays as unity, becomes 0·5 for superficial therapy, and 0·7 for the 200 kV range (Table II). In the case of deep-seated tumours treated with 200 kV radiation, the half-value layer (and hence the RBE) in the tissues differs from that of the incident beam on account of the increase in the average wave-length brought about by the Compton recoil process. This effect is the more marked the harder the incident radiation. From data by Stenstrom and Marvin (1946) it appears the HVL in the depths approaches 0·7 mm. Cu, irrespective of the quality of the incident ray. This corresponds (Fig. 2) to an RBE of 0·6.

The biological dosage (rec) was taken as equal to Dose/(RBE $\times$ $T^n$), where $n = 0{\cdot}25$ (as will be explained below a value of 0·25 gave the smallest variance, and is consequently considered the most accurate estimate). The column of results describes the behaviour of the tumours during the three years following irradiation. " Cured " implies a permanent local regression, irrespective of the patient's general health or the presence of metastases ; " recurred " implies a temporary response of the tumour followed by recurrence within the treated field ; where no regression has occurred the tumour is classified as " persisting " ; and in two cases in the higher dosage range the treated area " necrosed," accompanied by local recurrence.

For the purpose of correlating the probability of cure with dosage, the 100 cases were divided, in order of increasing dosage, into the five equal groups shown in Table III. The average dose received by each group was determined (on account of some outlying values the median dose was taken in preference to the mean), and the number of cured cases in the group was counted. In Fig. 3 each

TABLE III.—*Results of Treatment.*

| Case No. | Site. | Field (cm.). | RBE*. | Dose* (r). | Time (days). | Rec.* | Result. | Median dose. | Percentage cured. |
|---|---|---|---|---|---|---|---|---|---|
| 151 | Face | 5 ⊙ | 0·7 | 3200 | 60 | 1630 | Recurred | | |
| 152 | Orbit | 7×7 | 0·7 | 2300 | 7 | 1730 | ,, | | |
| 153 | Neck node | 15×15 | 0·6 | 2250 | 12 | 2010 | Persists | | |
| 154 | ,, ,, | 9 ⊙ | 0·6 | 3300 | 40 | 2180 | ,, | | |
| 155 | Larynx | 8×6 | 0·6 | 3150 | 32 | 2210 | ,, | | |
| 156 | Back | 3 ⊙ | 0·5 | 2000 | 10 | 2250 | ,, | | |
| 157 | Tongue | 10×8 | 0·6 | 3800 | 45 | 2430 | Recurred | | |
| 158 | Pharynx | 8×6 | 0·6 | 3500 | 23 | 2650 | ,, | | |
| 159 | Neck | 10×10 | 0·5 | 3000 | 21 | 2800 | ,, | | |
| 160 | ,, | 3 ⊙ | 0·5 | 1400 | 1 | 2800 | Persists | 2815 | 2/20 =10% |
| 161 | Nose | 3 ⊙ | 0·5 | 2000 | 4 | 2830 | Recurred | | |
| 162 | Alveolus | 8 ⊙ | 0·5 | 3000 | 18 | 2900 | Persists | | |
| 163 | Neck | 8×20 | 0·5 | 3150 | 22 | 2900 | ,, | | |
| 164 | Face | 10×10 | 0·5 | 3000 | 18 | 2900 | Recurred | | |
| 165 | Scalp | 14 ⊙ | 0·5 | 3300 | 26 | 2900 | ,, | | |
| 166 | Larynx | 8 ⊙ | 0·6 | 4400 | 40 | 2940 | Cured | | |
| 167 | Tongue, base | 15×10 | 0·6 | 3600 | 17 | 2950 | Persists | | |
| 168 | Pharynx | 4 ⊙ | 1·0 | 5000 | 8 | 2960 | ,, | | |
| 169 | Orbit | 7×7 | 0·6 | 3750 | 19 | 2990 | Cured | | |
| 170 | Face | 3 ⊙ | 0·5 | 2250 | 5 | 3000 | Persists | | |
| 171 | Temple | 4×4 | 0·5 | 3300 | 22 | 3050 | Recurred | | |
| 172 | Post-cricoid | 15×6 | 0·6 | 4600 | 40 | 3060 | Cured | | |
| 173 | Alveolus | 6×6 | 0·5 | 3700 | 33 | 3090 | Recurred | | |
| 174 | Face | 6×6 | 0·5 | 3000 | 14 | 3100 | ,, | | |
| 175 | Penis | 8×5 | 0·7 | 4200 | 14 | 3100 | Cured | | |
| 176 | Scalp | 14 ⊙ | 0·5 | 3300 | 20 | 3110 | Recurred | | |
| 177 | Epiglottis | 10×8 | 0·6 | 4600 | 36 | 3140 | ,, | | |
| 178 | Tonsil | 8×6 | 0·6 | 4300 | 26 | 3140 | ,, | | |
| 179 | Face | 3 ⊙ | 0·5 | 1600 | 1 | 3200 | Cured | | |
| 180 | Larynx | 7×7 | 0·6 | 4200 | 23 | 3200 | ,, | 3200 | 11/20 =55% |
| 181 | Epiglottis | 8×6 | 0·6 | 4300 | 26 | 3200 | ,, | | |
| 182 | Ear, pinna | 8 ⊙ | 0·5 | 3350 | 18 | 3250 | Recurred | | |
| 183 | Pharynx | 15×10 | 0·6 | 4000 | 18, | 3250 | Cured | | |
| 184 | Nasopharynx | 18×10 | 0·6 | 4000 | 18 | 3250 | ,, | | |
| 185 | Nose | 4×4 | 0·7 | 5000 | 23 | 3250 | ,, | | |
| 186 | Ear, pinna | 2 ⊙ | 0·5 | 2150 | 3 | 3260 | ,, | | |
| 187 | Scalp | 12×12 | 0·5 | 3450 | 20 | 3270 | ,, | | |
| 188 | Lip | 5 ⊙ | 0·5 | 3800 | 28 | 3300 | Recurred | | |
| 189 | Tonsil | 10×10 | 0·6 | 4100 | 18 | 3300 | ,, | | |
| 190 | Ear, pinna | 2×2 | 1·0 | 3300 | 1 | 3300 | Cured | | |
| 191 | Lip | 8×6 | 0·5 | 3600 | 22 | 3330 | Recurred | | |
| 192 | Scalp | 2 ⊙ | 0·5 | 2000 | 2 | 3350 | Cured | | |
| 193 | Face | 2 ⊙ | 0·5 | 2000 | 2 | 3350 | ,, | | |
| 194 | ,, | 4 ⊙ | 0·5 | 2550 | 5 | 3400 | ,, | | |
| 195 | ,, | 3 ⊙ | 0·5 | 1700 | 1 | 3400 | ,, | | |
| 196 | ,, | 3 ⊙ | 0·5 | 1700 | 1 | 3400 | ,, | | |
| 197 | Larynx | 8×6 | 0·6 | 4500 | 23 | 3420 | ,, | | |
| 198 | Cheek, oral | 5 ⊙ | 0·5 | 3300 | 13 | 3480 | ,, | | |
| 199 | Scalp | 10×10 | 0·5 | 4000 | 25 | 3560 | ,, | | |
| 200 | Hand, dorsum | 4 ⊙ | 0·5 | 2700 | 5 | 3600 | ,, | 3600 | 17/20 =85% |
| 201 | Face | 3 ⊙ | 0·5 | 1800 | 1 | 3600 | ,, | | |
| 202 | Penis | 4×4 | 0·5 | 3400 | 13 | 3600 | ,, | | |
| 203 | Scalp | 3 ⊙ | 0·5 | 3400 | 12 | 3620 | ,, | | |
| 204 | Lip | 7×7 | 0·7 | 3650 | 4 | 3630 | Recurred | | |
| 205 | Tongue | 7×7 | 0·6 | 4600 | 20 | 3630 | Cured | | |
| 206 | ,, | 7×7 | 1·0 | 7700 | 20 | 3640 | ,, | | |
| 207 | ,, | 10×10 | 0·6 | 4800 | 23 | 3650 | Recurred | | |
| 208 | Neck nodes | 18×10 | 0·6 | 4500 | 18 | 3650 | Cured | | |
| 209 | Nose | 3 ⊙ | 0·5 | 3350 | 11 | 3700 | ,, | | |
| 210 | Hand, dorsum | 6×6 | 0·5 | 3400 | 11 | 3750 | ,, | | |

TABLE III.—*cont.*

| Case No. | Site | Field (cm.). | RBE.* | Dose* (r). | Time (days). | Rec.* | Result. | Median dose. | Percentage cured. |
|---|---|---|---|---|---|---|---|---|---|
| 211 | Neck | 3 ⊙ | 0·5 | 3700 | 15 | 3750 | Cured | | |
| 212 | Lip | 4 ⊙ | 0·5 | 3500 | 12 | 3760 | „ | | |
| 213 | „ | 4 ⊙ | 0·5 | 3500 | 12 | 3760 | „ | | |
| 214 | Nose | 4 ⊙ | 0·5 | 4000 | 20 | 3800 | „ | | |
| 215 | Ear, back | 5 ⊙ | 0·5 | 3400 | 10 | 3810 | „ | | |
| 216 | Nose | 3 ⊙ | 0·5 | 3000 | 6 | 3840 | „ | | |
| 217 | Face | 3 ⊙ | 0·5 | 2550 | 3 | 3850 | „ | | |
| 218 | Branchial | 10×8 | 0·6 | 4500 | 13 | 3950 | „ | | |
| 219 | Alveolus | 7×7 | 0·6 | 5000 | 20 | 2980 | „ | | |
| 220 | Ear, pinna | 4×3 | 1·0 | 4000 | 1 | 4000 | „ | 4000 | 19/20 =95% |
| 221 | Hand, dorsum | 4 ⊙ | 1·0 | 6000 | 5 | 4000 | „ | | |
| 222 | Face | 3 ⊙ | 0·5 | 2000 | 1 | 4000 | „ | | |
| 223 | Tongue, base | 7×7 | 0·6 | 5600 | 29 | 4000 | Recurred | | |
| 224 | Lip | 4×4 | 0·6 | 4500 | 12 | 4050 | Cured | | |
| 225 | Neck node | 6×6 | 1·0 | 6400 | 6 | 4100 | „ | | |
| 226 | „ „ | 7×7 | 1·0 | 6500 | 6 | 4150 | „ | | |
| 227 | Nose | 3 ⊙ | 0·5 | 4000 | 13 | 4200 | „ | | |
| 228 | Ear, pinna | 2 ⊙ | 0·5 | 2100 | 1 | 4200 | „ | | |
| 229 | Hand, dorsum | 4×4 | 0·5 | 4500 | 17 | 4300 | „ | | |
| 230 | Tongue | 5×5 | 1·0 | 7000 | 7 | 4300 | „ | | |
| 231 | Lip | 4 ⊙ | 0·5 | 4000 | 21 | 4300 | Cured | | |
| 232 | Ear, pinna | 5×2 | 0·5 | 4100 | 13 | 4300 | „ | | |
| 233 | Face | 3 ⊙ | 0·5 | 3400 | 5 | 4500 | „ | | |
| 234 | Lip | 5 ⊙ | 0·5 | 4200 | 12 | 4510 | „ | | |
| 235 | Larynx | 4×4 | 0·6 | 9500 | 144 | 4550 | „ | | |
| 236 | Hand, dorsum | 8 ⊙ | 0·5 | 5000 | 23 | 4600 | Necrosed | | |
| 237 | Neck | 3 ⊙ | 0·5 | 4000 | 9 | 4600 | Cured | | |
| 238 | Face | 5 ⊙ | 0·5 | 4250 | 11 | 4700 | „ | | |
| 239 | Antrum | 6 ⊙ | 1·0 | 8000 | 8 | 4750 | „ | | |
| 240 | Ear, pinna | 8 ⊙ | 0·5 | 5200 | 21 | 4850 | Necrosed | 4850 | 17/20 =85% |
| 241 | Temple | 3 ⊙ | 0·5 | 4200 | 9 | 4850 | Recurred | | |
| 242 | Face | 3 ⊙ | 0·5 | 3800 | 5 | 5000 | Cured | | |
| 243 | Cheek | 2 ⊙ | 0·5 | 2500 | 1 | 5000 | „ | | |
| 244 | Face | 3×2 | 1·0 | 6000 | 2 | 5050 | „ | | |
| 245 | Cheek | 4×4 | 0·5 | 5200 | 15 | 5200 | „ | | |
| 246 | Face | 3 ⊙ | 0·5 | 5100 | 13 | 5310 | „ | | |
| 247 | Nose | 3 ⊙ | 0·5 | 6000 | 16 | 6000 | „ | | |
| 248 | Scalp | 8 ⊙ | 0·5 | 6000 | 16 | 6000 | „ | | |
| 249 | Forehead | 3 ⊙ | 0·5 | 6000 | 14 | 6200 | „ | | |
| 250 | Face | 2×2 | 0·5 | 5100 | 7 | 6200 | „ | | |

* RBE = "Reciprocal biologic efficiency" compared to $\gamma$-rays as unity.
Dose = Minimum tumour dose, estimated for each case from isodoses.
Rec = "Roentgen equivalent clinical"; dose corrected for quality and time.

step represents one group of 20 cases. The horizontal extent of the step illustrates the range of dosage, while the vertical height indicates the percentage of tumours cured in that group. The circles represent the median dose for each group, and the smooth curve drawn through them is probably the true curability curve for epidermoid cancer. It is of interest to note that in the highest dosage range the curability is considerably less than expectation. This deviation is associated with the cases of "necrosis plus recurrence," and confirms the existence of the supralethal effect (Paterson, 1948).

In Fig. 4 the same data are charted on lognormal probability co-ordinates, the ordinate representing the percentage cured. The straight line obtained proves the almost perfect lognormality of the grouped data. The mean lethal

FIG. 3.—Cumulative frequency diagram illustrating increased proportion of tumours cured with increasing biological dosage. Doses are expressed in roentgens equivalent clinical (rec).

FIG. 4.—Test for lognormality of the tumour dosage data. The outlying point on the right illustrates the "supralethal effect." The inset demonstrates a method for determining the recovery exponent (n).

dose is seen to be about 3000 rec (in good agreement with previous estimates), and the uncertainty factor is 1·3.

The inset in Fig. 4 illustrates a method for determining the recovery exponent (n) by a graphical solution. Various values of n ranging from zero to 0·5 were selected, and the resultant estimates of biological dose tabulated in a manner

Fig. 5.—Nomogram relating skin tolerance and epidermoid cancer lethal doses with time and volume factors. Doses apply to conventional 200 kV deep therapy. A single line across the diagram gives all the relevant factors simultaneously.

similar to Table III but regrouped accordingly. The cure rates so obtained were similarly charted in lognormal probability graphs, and the values of the standard deviation factor (f) corresponding to each value of n was determined. It is seen that f is a minimum as n approximates 0·25. The disadvantage of this graphical method, which is otherwise simple and sufficiently accurate for practical purposes, is that it furnishes no means for computing the variance of n. It is, therefore, impossible to test the agreement between our rough estimate of n = 0·25

and similar data from other sources, e.g., Strandqvist's (1944) n = 0·22. However, Kerrich (Statistical Note) estimates this value to lie between 0·24 and 0·34, with a mean of 0·29.

### DISCUSSION.

*Therapeutic Ratio.*

The object of this investigation is to determine the optimum dose for the treatment of epidermoid cancer. For this purpose it is necessary to deliver that dose which will give the largest proportion of tumour regressions, and yet rarely or never exceed the limits of skin tolerance. It has been shown that a standard erythema reaction is given in the average case by 1000 rec, and in 95 per cent of cases the dose lies between $\pm$ 25 per cent of this value (that is, 800 to 1250 rec). An erythema would be encountered in less than 2 per cent of cases receiving 750 rec or lower ; and the same proportion of cases, therefore, might be expected to develop necrosis with 3000 rec. Estimated on the basis of the iso-effect formula, then 3000 rec can be accepted as the skin tolerance dose, with a risk of necrosis of under 2 per cent.

The probability of curing an irradiated tumour increases with increasing dosage up to a maximum of 95 per cent at a dose of 4000 rec (Fig. 3), beyond which the supralethal effect becomes increasingly menacing. It is, therefore, advisable to exceed the mean lethal dose of 3000 rec whenever possible by a factor approaching two standard deviations (30 per cent), remaining, of course, within the bounds of the skin tolerance.

The ratio of skin tolerance to mean lethal dose has been defined as the therapeutic ratio ; and the writer has shown (Cohen, 1949) that this ratio can be varied within wide limits by a judicious selection of field-size and over-all time. It was pointed out that the therapeutic ratio must always be greater than unity and, if the radiation is not absolutely homogeneous, should exceed the heterogeneity factor. When the therapeutic ratio equals 1·00, the skin tolerance dose is identical to the mean tumour lethal dose, so that the MLD cannot be exceeded, and a cure rate of not more than 50 per cent can be expected. It has been shown that the maximum probability of cure is given by a tumour dose of 1·3 MLD's. In general, therefore, the optimum combination of time and area is that which gives a therapeutic ratio of not less than 1·3, in which case the probability of cure may approach 95 per cent. The time and area factors corresponding to a given therapeutic ratio can be determined from the author's nomogram (Cohen, 1949), of which a modified version is reproduced in Fig. 5.

It has been asserted that the prognosis is proportional to the therapeutic ratio. Since the probability of cure is dependent both on this ratio and on the variance of the MLD, the statement can be expressed quantitatively in the form

$$\theta = \mathrm{H}.\mathrm{f}^{c}, \qquad \text{or} \qquad c = \frac{\log \frac{\theta}{\mathrm{H}}}{\log \mathrm{f}},$$

where $\theta$ is the therapeutic ratio obtaining with a given combination of field-size and treatment time, H is the heterogeneity factor, equal to the ratio of the maximum tissue dose to the minimum tumour dose, and f is the standard deviation factor, equal to 1·15 for our data.

It is then possible to calculate c the normal deviate, with which is associated a definite probability. The probability of cure, corresponding to any given value of c, can be determined from statistical tables or from the ordinates of Fig. 4. It will be noted that c = 0 corresponds to a cure rate of 50 per cent ; c = 1 to 85 per cent ; and c = 2 to 97·5 per cent. In this way a definite prognosis, as far as the primary growth is concerned, can be assigned to any epithelioma of known size treated, over a given number of days, to the limits of skin tolerance.

SUMMARY.

A statistical analysis of the intensity of the skin reaction in 150 cases, and its dependence on the time (T), field-size (L), and the biological efficacy (a) of the radiation used, confirmed the general validity of the simplified "iso-effect" formula $D = E.a.\left(\frac{T}{L}\right)^n$. By means of the method of least squares, estimates of a and n for six different qualities of radiation were obtained, and shown to be in good agreement with previously established data. The uncertainty factor in dosage estimated on this basis ranged from 1·21 to 1·35.

A further 100 cases of epidermoid cancer were similarly analysed in order to determine the dependence of curability on the biological dose. The median lethal dose for this tumour (3000 rec) and its uncertainty factor (1·3) were determined, and it was shown that, under optimal conditions of time and area, 95 per cent of tumours could be cured with a dose exceeding the median lethal dose by 30 per cent. It has also been demonstrated that the prognosis is a mathematical function of the therapeutic ratio.

The author wishes to acknowledge the valuable advice given by Mr. J. E. Kerrich, Department of Mathematics, University of the Witwatersrand, and his generous assistance with the statistical portion of this work. In particular he has examined the graphical method for determining the recovery exponent (n) for epidermoid cancer, and considers the operation mathematically legitimate and reasonably accurate.

STATISTICAL NOTE BY J. E. KERRICH.

From the data in Table III Dr. Cohen suggests that biological dose = dose/RBE × $T^n$, or $X = Z/T^n$ say, where X = biological dose, Z = dose/RBE, T = time in days. He goes on to show that if X is the exact dose necessary to effect a cure, then a reasonable assumption is that $\log X = \log Z - n \log T$ is normally distributed.

Writing $x = \log X$, $z = \log Z$, $t = \log T$ this hypothesis becomes $x = z - nt$ is normally distributed with, say, mean $\alpha$ and variance $\sigma^2$.

By a neat graphical method he proceeds to estimate $\alpha$, n and $\sigma$. The disadvantage of his method is that it affords no clue as to the reliability of his estimates. To study this question of reliability we must employ an algebraic method. The author's problem is, in principle, reminiscent of a problem examined in D. J. Finney's *Probit Analysis* (p. 106, Ex. 18), and adapting the algebraic methods explained in Appendix II of that book, the following results were obtained (omitting cases 236 and 240) :

| | $\alpha$ | n | $\sigma$ |
|---|---|---|---|
| Estimated value . . . | 3·4642 | ·2893 | ·0709 |
| Estimated standard deviation | ·0359 | ·0285 | ·0186 |

The sizes of the estimated standard deviations show that none of the constants can claim to be very "well determined." It must, however, be remembered that we are not dealing with a planned experiment, but that the author had to use the data that were to hand. As a suggestion to future workers in this field, more observations for small values of t (say t = 0) and large values of t (say t = 1·5) would help greatly in fixing $\alpha$ and n more accurately.

In more detail, the estimated covariance matrix is—

$$\begin{array}{lll} \text{var.}\ (\alpha) = \cdot 001292 & \text{cov}\ (\alpha, n) = -\cdot 000955 & \text{cov}\ (\alpha, \sigma) = -\cdot 000383 \\ & \text{var}\ (n) = +\cdot 000813 & \text{cov}\ (n, \sigma) = +\cdot 000227 \\ & & \text{var}\ (\sigma) = \cdot 000348 \end{array}$$

The mean value of x is $\alpha$. We estimate that this lies between $3{\cdot}4642 \pm {\cdot}0718$ with 5 per cent risk. This makes our estimate of median X to be 2912 rec with a 5 per cent uncertainty factor of 1·180.

If x is greater than $\alpha + 2\sigma$, then the cure rate should be 97·5 per cent or more. Our estimate of $\alpha + 2\sigma$ is $3{\cdot}6060 \pm {\cdot}0679$, and the corresponding biological dose is X = 4036 rec with a 5 per cent uncertainty factor of 1·169.

Our thanks are due to the computing staff of the National Institute of Personnel Research for their help in preparing this note.

### REFERENCES.

COHEN, L.—(1949) *Brit. J. Radiol.*, **22**, 160, 706.
GRYNKRAUT, B., AND SITKOWSKI, J.—(1936) *Strahlentherapie*, **56**, 413.
HELMKE, R.—(1949) *Ibid.*, **80**, 585.
JOLLES, B.—(1950) *Brit. J. Radiol.*, **23**, 18.
MACCOMB, W. S., AND QUIMBY, E. H.—(1937) *Radiology*, **29**, 305.
MACKEE, G. M., AND CIPOLLARO, A. C.—(1940) *Arch. Derm. Syph.*, **41**, 1.
PATERSON, R.—(1948) 'The Treatment of Malignant Disease by Radium and X-rays.' London (Arnold & Co.), p. 171.
QUASTLER, H., AND CLARK, R. K.—(1945) *Amer. J. Roentgenol.*, **54**, 723.
SIEVERT, R. M.—(1947) *Brit. J. Radiol.*, **20**, 306.
SUGIURA, K.—(1939) *Amer. J. Cancer*, **37**, 445.
STENSTROM, K. W., AND MARVIN, M. S.—(1946) *Amer. J. Roentgenol.*, **56**, 759.
STRANDQVIST, M.—(1944) *Acta Radiol.*, Suppl. 55, 1.
TOD, M.—(1950) *J. Faculty Radiol.*, **2**, 43.